# Optimization of Breast Tomosynthesis Visualization through 3D Volume Rendering

**DOI:** 10.3390/jimaging6070064

**Published:** 2020-07-04

**Authors:** Ana M. Mota, Matthew J. Clarkson, Pedro Almeida, Nuno Matela

**Affiliations:** 1 Instituto de Biofísica e Engenharia Biomédica, Faculdade de Ciências, Universidade de Lisboa, 1749-016 Lisboa, Portugal; palmeida@fc.ul.pt (P.A.); nmatela@fc.ul.pt (N.M.); 2 Department of Medical Physics and Biomedical Engineering and the Centre for Medical Image Computing, University College London, London WC1E 6BT, UK; m.clarkson@ucl.ac.uk

**Keywords:** breast tomosynthesis, visualization, volume rendering

## Abstract

3D volume rendering may represent a complementary option in the visualization of Digital Breast Tomosynthesis (DBT) examinations by providing an understanding of the underlying data at once. Rendering parameters directly influence the quality of rendered images. The purpose of this work is to study the influence of two of these parameters (voxel dimension in *z* direction and sampling distance) on DBT rendered data. Both parameters were studied with a real phantom and one clinical DBT data set. The voxel size was changed from 0.085 × 0.085 × 1.0 mm^3^ to 0.085 × 0.085 × 0.085 mm^3^ using ten interpolation functions available in the Visualization Toolkit library (VTK) and several sampling distance values were evaluated. The results were investigated at 90º using volume rendering visualization with composite technique. For phantom quantitative analysis, degree of smoothness, contrast-to-noise ratio, and full width at half maximum of a Gaussian curve fitted to the profile of one disk were used. Additionally, the time required for each visualization was also recorded. Hamming interpolation function presented the best compromise in image quality. The sampling distance values that showed a better balance between time and image quality were 0.025 mm and 0.05 mm. With the appropriate rendering parameters, a significant improvement in rendered images was achieved.

## 1. Introduction

Breast cancer remains the most common cancer diagnosed among women and a leading cause of death. However, in the last three decades, there has been a decrease of approximately 40% in the death rate from this disease [1,2]. This fact is a direct result of the scientific advances in early detection and treatment. Early detection is mostly done through screenings [3,4]. Until recently, these screenings and breast cancer diagnosis in general were mainly performed by Digital Mammography (DM). However, DM consists of a two-dimensional (2D) acquisition of the three-dimensional (3D) breast causing tissue superposition. This often results in malignant lesions hidden between healthy tissues or normal regions considered as pathological, leading to unnecessary second examinations or biopsies, with additional costs and anxiety for patients [5,6].

Digital Breast Tomosynthesis (DBT) has consolidated its position as a technique to replace DM in both screening and clinical environments [7,8,9,10,11]. DBT has an acquisition geometry very similar to DM but it acquires a set of projection images, allowing a 3D reconstruction of the breast, reducing the tissue overlap observed with DM [12]. In this way, DBT improves the perception of the location and shape of lesions, without increasing the radiation dose to the patient, when compared to DM [13].

The 3D visualization of DBT is one of the most important and crucial aspects to correctly extract the information provided by this technique. Currently, DBT images are displayed through a 2D slice-by-slice visualization [14], with the analysis done one slice at a time or sequentially as a continuous cine loop, leading to a time-consuming process. Two other new approaches have emerged for the visualization of DBT data: synthetic mammography based on DBT data [15,16] and thicker slabs obtained by combining several slices [17,18]. Although synthetic mammography is very useful for comparison with previous DM examinations, it still presents the disadvantages of a 2D visualization (tissue overlapping) [19]. On the other hand, the thicker slabs have revealed good results in reducing time and false positives but have a lower sensitivity [18]. Additionally, a system very recently approved by the Food and Drug Administration, uses artificial intelligence to reduce the number of slices that need to be analyzed by the radiologist. It is based on thicker 6 mm slices combined with synthesized 2D images information [20,21].

A different type of visualization may play an important complementary role in breast cancer diagnosis [22]. 3D volume rendering is the process of creating realistic computer-generated images of volumetric data, yielding a true depth perception [23]. This type of supplementary visualization is already used in other tomographic medical imaging modalities such as Computed Tomography, Magnetic Resonance Imaging, or Positron Emission Tomography [24,25,26]. There are some works mentioning a few aspects of 3D volume rendering for DBT [27,28,29] and its importance to detect clusters of microcalcifications [30].

The classic volume rendering algorithm is ray casting, where rays are cast from the eye or other viewpoints and traverse a scene containing a volumetric data set. This process encompasses several parameters that decide the appearance of the final rendered image. One of these parameters is the sampling distance, which corresponds to the distance between neighboring samples taken along the ray. The value of the sampling distance should be studied and carefully selected accordingly to data set grid resolution [31]. If the distance is too large, our sampling might miss important features in the data and generate major aliasing artifacts. Yet, if we select a very small distance (the number of samples collected along the ray is increased), the amount of time required to render the image will significantly increase [32,33]. 

On the other hand, reconstructed DBT data typically has voxel sizes of 0.085 × 0.085 × 1.0 mm^3^. The anisotropic nature of the reconstructed DBT data is also responsible for serious quality problems in visualization techniques, namely in the direction orthogonal to the detector plane (z-direction). In this way, one solution is to make the grid isotropic through suitable interpolation functions before the rendering process [31]. The smaller the voxel size, the higher the image definition. However, there are more voxels compounding the data set and therefore the processing time of each data set is longer. The time issue is very important in medical image analysis because a large amount of data needs to be displayed and analyzed in real time. For this study, we consider the total time allocated to the visualization process as the sum of interpolation time with rendering time.

In this paper, two parameters that directly affect the quality of the rendered image—sampling distance and reconstructed voxel size—were considered. The main objective was to improve the quality of the rendered images in the *z* direction and to determine which options allow a better balance between quality and time. In order to transform data to an isotropic grid, several interpolation functions and their corresponding parameters were tested. Additionally, different sampling distance values were introduced in the rendering process. Qualitative and quantitative analyzes of the results were done through visualization by volume rendering of real DBT images of a phantom. Finally, some values were selected and the rendered images obtained for one clinical DBT data set were also analyzed. To the best of our knowledge, this is the first study about optimization of rendering parameters in visualization of DBT data.

## 2. Materials and Methods

### 2.1. Data Acquisition and Reconstruction

**Figure 1:**
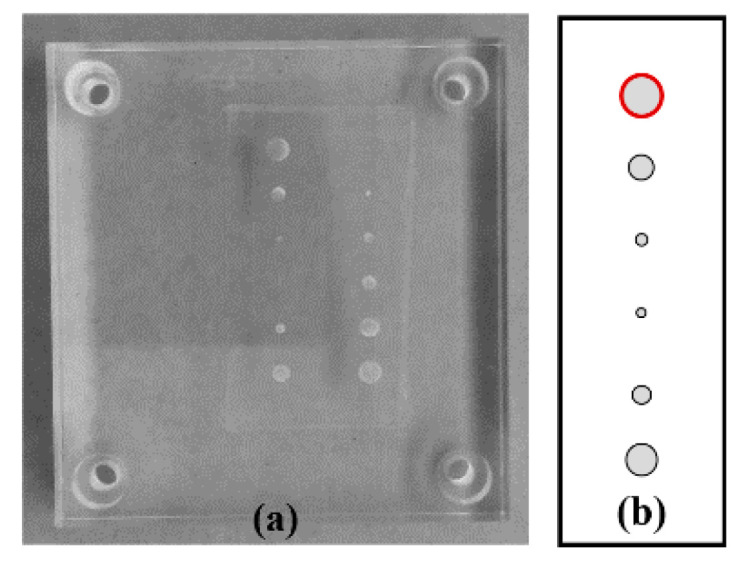
(**a**) Acrylic phantom simulating breast tissue and high density lesions (aluminum disks of different diameters and 1 mm thickness). (**b**) Scheme of the disks in the first column (top to bottom): 5.0 mm, 3.0 mm, 1.0 mm, 0.5 mm, 2.0 mm, and 4.0 mm, respectively.

A phantom made by us was used (Figure 1). This phantom consists of an acrylic background to mimic breast tissue, with two embedded columns of aluminum disks to simulate high density lesions (with different diameters and 1.0 mm thick). For this study, the first column of the phantom (Figure 1b) was considered.

Additionally, one clinical DBT data set from an anonymous patient was selected from the clinical facility (Hospital da Luz, Luz Saúde S.A, Lisbon, Portugal) database. Both phantom and clinical data set were acquired with a Siemens MAMMOMAT Inspiration system (Siemens AG, Healthcare Sector, Erlangen, Germany) and reconstructed with the manufacturer algorithm, which uses Filtered Back Projection [34]. The reconstructions have voxel sizes of 0.085 × 0.085 × 1.0 mm^3^.

### 2.2. Data Visualization

**Figure 2:**
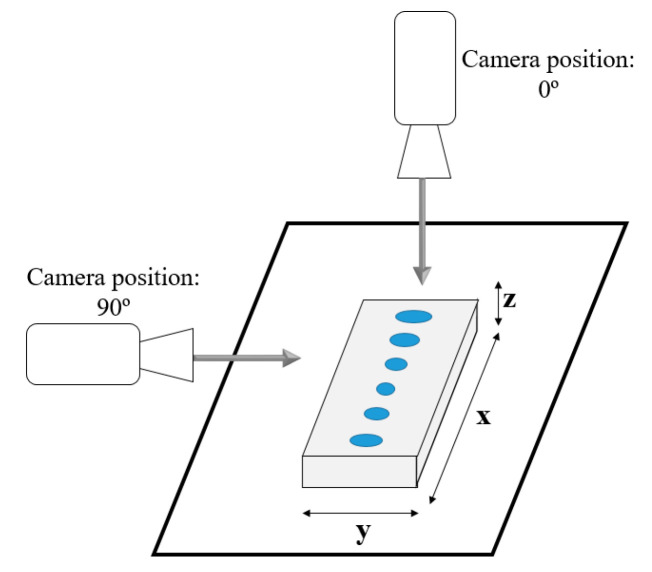
Illustrative scheme of visualization at 0° and 90°.

In volume rendering, changing the azimuth of a camera rotates its position around the focal point [32]. In this way, it is possible to have an immediate notion of the entire data volume, according to several angles. The methods under study in this work have a particular effect on the z-direction. Therefore, we were particularly interested in the visualization perpendicular to the detector plate (with the projection made on the *xz* planes, along *y*), i.e., with the camera at 90º (see Figure 2). In addition, the visualization with the camera at 0°, that is, parallel to the detector plate (projection is made on the *xy* planes, along *z*) was also considered.

The Visualization Toolkit library (VTK) version 7.1.0. (Kitware, Clifton Park, NY, USA) [32,35] was used to develop 3D specific software in order to visualize DBT data through volume rendering. The methodologies in study were analyzed using 3D volume rendering visualization with composite technique. An Intel^®^ Core ™ i5-5200U CPU (2.20 GHz) @ 8 GB of memory computer was used.

### 2.3. Image Analysis

For phantom quantitative analysis, the profile of the 5.0 mm disk (Figure 1b in red) was obtained and three figures of merit were used: full width at half maximum (FWHM) of a Gaussian curve fitted to the 5.0 mm disk’s profile, contrast to noise ratio (CNR), and a measure of profile smoothness. FWHM_90º_ was considered as an indicator of the disk’s definition at 90°. As already mentioned, DBT presents a lower quality in *z* than in *xy* and this is exposed in the spreading of structures of high intensity in *z* (such as these disks). Although the disks have a thickness of 1.0 mm, the observed values will be higher. In this way, a lower value of FWHM_90º_ will mean a greater definition of the feature at 90°. For CNR, a region of interest (ROI) over the 5.0 mm disk and other two ROIs over the surrounding background were drawn. CNR was calculated using Equation (1):

(1) $$CNR=\frac{\mu_{5.0\;mm}-\mu_{BG}}{\sigma_{BG}}$$

$\mu_{5.0mm}$ and $\mu_{BG}$ stand for mean pixel values in ROI over the 5.0 mm disk and background, respectively; and *σ_BG_* stands for the mean of standard deviations in background ROIs. In order to obtain a measure for the profile smoothness (important for the interpolation quality analysis), the *STEYX* Microsoft Excel^®^ (Microsoft Office 2013) function was used [36] and its inverse was calculated (Equation (2)).The *STEYX* function gives a measure of the variability of the data in a given range. The degree of smoothness was calculated considering intensity levels between z-distance (16, 24) mm since it corresponded to an area with high variation in intensity:

(2) $$Smoothness=\frac{1}{STEYX_{\left[16,24\right]mm}}$$

The quantitative analysis was performed at 90°, where the methods under study have the greatest effect. For qualitative purposes, displays of the phantom and one clinical data set obtained with volume rendering visualization at 0° and 90° are presented.

### 2.4. Study of Interpolation Functions

Due to the acquisition process, DBT data has a finer resolution within slices (in *xy* planes) and a coarser resolution between slices (in the *z*-direction). This leads to an anisotropic grid, greatly reducing the quality of rendering. To deal with the anisotropic grid spacing problem, one hypothesis is to change the size of each voxel, considering the smallest dimension, so it can correspond to a perfect cube [37]. Therefore, in this study, the z-resolution has been modified to match the resolution within the slice (which has not changed). This results in a homogeneous resolution, improving data quality after reconstruction and before rendering. At the same time, the number of voxels compounding the data set is also increased and thus more computational memory is required. We can refer to a z-interpolation, since the voxel size went from 0.085 × 0.085 × 1.0 mm^3^ to 0.085 × 0.085 × 0.085 mm^3^, that is, the change occurred only in the third dimension. 

As this work is based on C++ software developed with the VTK library, the volume data was resampled into an isotropic grid using appropriate interpolation functions available in this library [38]. In VTK, data interpolation is done internally by several classes. The two main classes considered here were *vtkImageInterpolator* and *vtkImageSincInterpolator*. The first one is the default interpolator and provides linear, cubic, and nearest-neighbor interpolation. The second is responsible for an approximation to sinc interpolation by multiplying one of the available window functions, in order to limit the kernel size. The window functions studied in this work were: Lanczos, Kaiser (adjusmet parameter off), Cosine, Hann, Hamming, Blackman, and Nuttall [38,39]. In Fourier space, the resolution of spectral window is reduced to the order of the half-width of the sinc function [40]. In this way, the quality of sinc interpolation is related not only to the window function, but also to the window width. For this reason, the window half-width (WHW) was also addressed in this study with values ranging between 1 and 16. *vtkImageSincInterpolator* class has an option to set blur factors in *x*, *y,* and *z* directions in order to blur the data while interpolating [38]. The size of the kernel is automatically increased by the blur factor (BF), increasing the computation time. As here the interpolation is in the z-direction, some values for BF in *z* were tested. A summary of the functions and parameters analyzed during the interpolation process is shown in Table 1.

For the interpolation study, the default value of sampling distance (1.0 mm) was used.

### 2.5. Study of Sampling Distance 

In the ray casting volume rendering algorithm, a ray traverses a volume data set. Along the ray, contributions (samples) based on the intensity values weighted by transparency or opacity (transfer functions) are accumulated at discrete locations of the ray, separated by a certain distance—sampling distance. The process of selecting these locations and, therefore, the distance between them, is subject to the sampling theorem. In this case, it is translated on the condition that the distance between two accumulations must be less than or equal to twice the respective smallest voxel spacing [31]. 

Taking into account the best results obtained with the interpolation functions and their parameters, several sampling distance values were studied. Considering the sampling theorem, values smaller than 2 × 0.085 mm (0.170 mm) were tested. As previously mentioned, the lower the sampling distance value, the higher the rendering quality should be. As the computation time increases exponentially for lower sampling distance values, our aim was to test several values smaller than 0.170 mm and try to understand where the best balance between quality and time could be achieved. In addition, five other values outside this range were tested. The default value for sampling distance in VTK is 1.0 mm and it was the maximum value included in our study. Included in the class corresponding to volume ray casting, VTK has an option which automatically computes the sampling distance from the data spacing. Using this option, an automatic sampling distance of 0.195 mm was generated. This value was also included in this work (from a brief previous study, it was concluded that, for our DBT data, this automatically generated value was related to the number of voxels according to an approximation of the expression autoSD = 3580.5 × (nr voxels)^(−0.621)^). On the other hand, in order to contextualize the results between 0.195 mm and 1.0 mm, some intermediate values (0.40 mm, 0.60 mm, and 0.80 mm) were also considered. In summary, the sampling distance values analyzed were: 0.010 mm, 0.025 mm, 0.050 mm, 0.075 mm, 0.100 mm, 0.145 mm, 0.170 mm (2 × 0.085 mm), 0.195 mm (automatic Sampling distance), 0.4 mm, 0.6 mm, 0.8 mm, and 1.0 mm (default value in VTK).

## 3. Results

**Figure 3:**
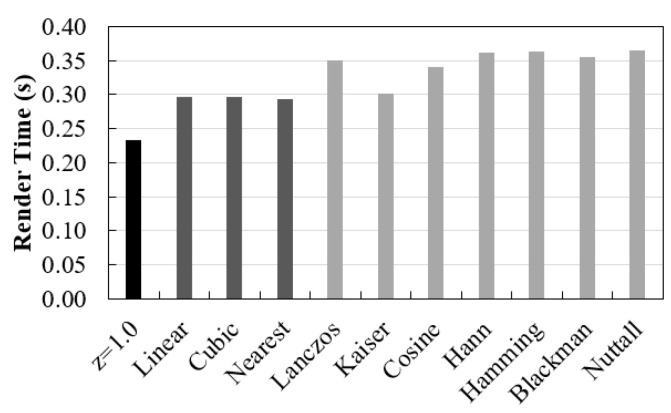
Computation time required for rendering the original data (z: 1.0 mm) and data after rescaling with linear, cubic, nearest interpolators and Lanczos, Kaiser, Cosine, Hann, Hamming, Blackman, and Nuttal window functions (with sampling distance 1.0 mm).

As mentioned, the time involved to render data is very important in order to make visualization through volume rendering useful. Here, we have separated total time spent in visualization as interpolation time plus render time (from the moment the original data is opened until it reaches the screen). The first depends on voxel size and interpolation functions used in the rescaling. The second is related with ray casting process, namely the sampling distance value. Render time values recorded for rendering the original data, as well as data after interpolation (with sampling distance 1.0 mm) are shown in Figure 3. 

As the interpolation was done only in the third dimension, all the results presented in this section were measured considering phantom visualization at 90° (i.e., through the *z* direction). 

### 3.1. Study of Image Interpolators

#### 3.1.1. Linear, Cubic, and Nearest-Neighbor Interpolation

**Figure 4:**
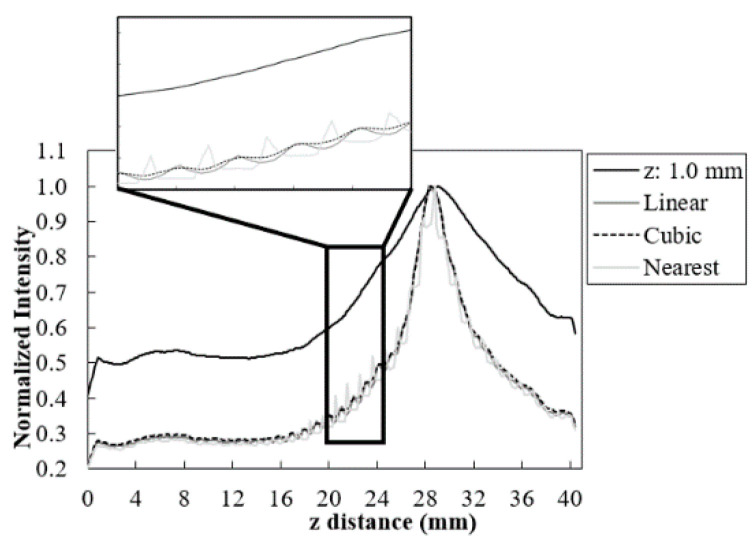
Profile of the 5.0 mm disk obtained at 90° for the original data (z: 1.0 mm) and after rescaling with the linear, cubic, and nearest interpolators. Zoom-in of a range with large intensity variation.

The 5.0 mm disk’s profiles obtained after rescaling with the linear, cubic, and nearest-neighbor interpolators are presented in Figure 4. Additionally, it is also shown the profile obtained before rescaling (with voxel size of 0.085 × 0.085 × 1.0 mm^3^). FWHM and smoothness values measured for each visualization at 90° are presented in Table 2. The total time required in the process is also shown and it stands for the rendered time (Figure 3) plus interpolation time. 

#### 3.1.2. Sinc Interpolation with Different Window Functions

**Figure 5:**
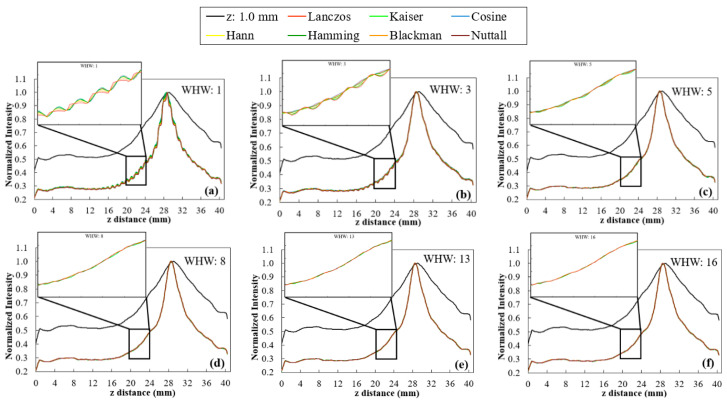
Profiles of the 5.0 mm disk obtained at 90º for the original data (z: 1.0 mm) and after rescaling with Lanczos, Kaiser, Cosine, Hann, Hamming, Blackman, and Nuttall window functions with WHW values of 1, 3, 5, 8, 13, and 16 ((**a**) to (**f**), respectively). Zoom-in of a range with large intensity variation.

The sinc function was multiplied by different window functions (Lanczos, Kaiser, Cosine, Hann, Hamming, Blackman, and Nuttall). The 5.0 mm disk’s profiles obtained with WHW values ranging from 1 to 16 are presented in Figure 5.

**Figure 6:**
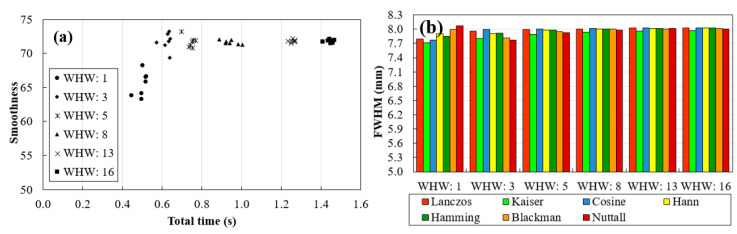
Results obtained with Lanczos, Kaiser, Cosine, Hann, Hamming, Blackman, and Nuttall window functions for the profile at 90°, by changing WHW values (from 1 to 16). (**a**) Smoothness values as a function of total time and (**b**) FWHM of the 5.0 mm disk at 90°.

Based on profiles of Figure 5 and for each window function and WHW, the corresponding smoothness and FWHM values were determined and the results are displayed in Figure 6.

**Figure 7:**
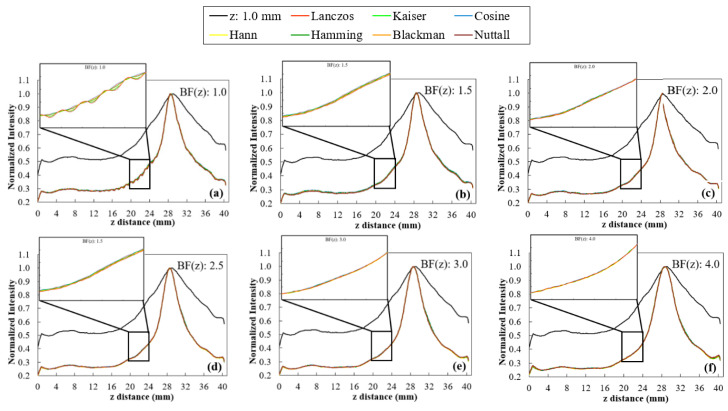
Profiles of the 5-mm disk obtained at 90° for the original data (z: 1.0 mm) and after rescaling with Lanczos, Kaiser, Cosine, Hann, Hamming, Blackman, and Nuttall window functions with BF(z) values of 1.0, 1.5, 2.0, 2.5, 3.0, and 4.0 (**a**–**f**). Zoom-in of a range with large intensity variation.

**Figure 8:**
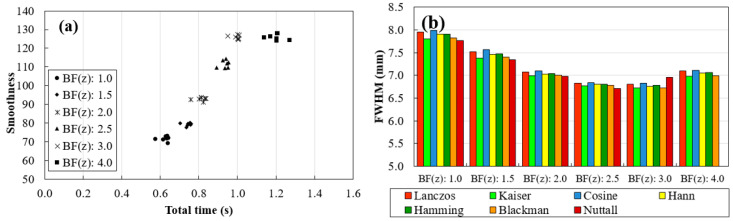
Results obtained with Lanczos, Kaiser, Cosine, Hann, Hamming, Blackman, and Nuttall window functions for the profile at 90°, by changing BF(z) values (from 1.0 to 4.0). (**a**) Smoothness values as a function of total time and (**b**) FWHM of the 5.0 mm disk at 90°.

The profiles calculated with BF (z) values ranging from 1.0 to 4.0 are shown in Figure 7 from (a) to (f), respectively. As for the WHW, based on profiles of Figure 7, the corresponding smoothness and FWHM values for each BF (z) and window function were determined and the results are presented in Figure 8.

### 3.2. Selection of Functions and Parameters for the Sampling Distance Study

The study of interpolation functions and their parameters is, by itself, quite extensive. Thus, to simplify the analysis of the results obtained with different sampling distance values, some selections were made to proceed.

- WHW: In Figure 5, for WHW values above 5, the obtained profiles are very similar. This is translated into the results of Figure 6a, where WHW = 5 produces the highest smoothness in the shortest time. From this value on, there is no significant increase in smoothness, only an increase in the time required for interpolation. On the other hand, in Figure 6b, the FWHM values are very similar for the different WHW values. In this way, the choice is based on the smoothest profile in the shortest possible time. This corresponds to WHW = 5.
- BF(z): According to Figure 7, for BF (z) ≥ 1.5, there is a significant decrease in the variability of the profiles. Through the results in Figure 8a, it is possible to observe that the higher the value of BF (z), the greater the smoothness, reaching a certain convergence for BF (z) ≥ 3. From BF (z) = 1.0 to BF (z) = 2.0, there is a visible decrease in the value of FWHM (Figure 8b), and for BF (z) > 2, these values become very similar. Thus, we choose BF (z) = 2 as the value that represents a better compromise between smoothness, FWHM, and time.
- Interpolator: Nearest and linear interpolators were excluded since the corresponding profiles showed low smoothness when compared to the others. The cubic interpolator was selected to proceed as it presented smoothness and FWHM values comparable to the other interpolations with a similar interpolation time. For the sinc interpolator, considering the results with WHW = 5 and BF (z) = 2, window functions were sorted according to the profile smoothness (in decreasing order). The Hamming window function presented the best correspondent result between the two options (for WHW = 5: Kaiser > Nuttall > Hamming; for BF (z) = 2: Blackman > Lanczos > Hann > Hamming). Since, among them, window functions presented very close results, this selection of a single function to proceed was done only to simplify and concentrate the next results.

In summary, the next section results are for cubic interpolation and Hamming window function (sinc) with WHW = 5 or/and BF(z) = 2.

### 3.3. Sampling Distance Study

**Figure 9:**
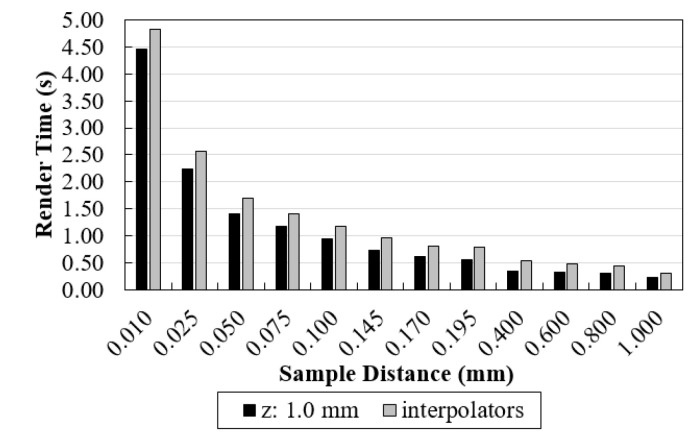
Computation time required for rendering the original data (black) and data after rescaling (gray) taking into account the different sampling distance values. For each sampling distance, each gray value was obtained by averaging the rendering times recorded for each interpolator considered here (cubic, Hamming with WHW = 5, Hamming with BF (z) = 2 and Hamming with WHW = 5 and BF (z) = 2).

For each sampling distance, the time required to render the original data (z: 1.0 mm, black in Figure 9) was recorded. In addition, the average rendering times of each selected interpolator (cubic, Hamming with WHW = 5, Hamming with BF (z) = 2 and Hamming with WHW = 5 and BF(z) = 2) were also calculated (gray in Figure 9).

**Figure 10:**
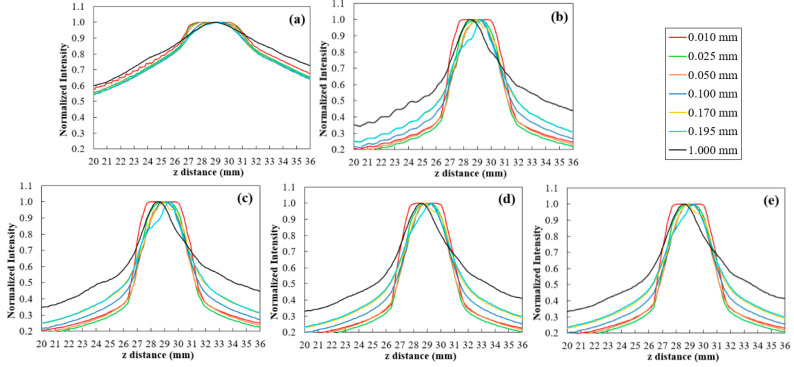
Profiles of the 5.0 mm disk obtained at 90°, for some values of sampling distance tested (0.010, 0.025, 0.050, 0.100, 0.170, 0.195, and 1.0 mm), with the original data (**a**) and after rescaling with cubic (**b**), Hamming with WHW = 5 (**c**), Hamming with BF (z) = 2 (**d**), and Hamming with WHW = 5 and BF (z) = 2 (**e**).

The 5.0 mm disk’s profiles were obtained from the rendered images with each sampling distance value and for each interpolator. The measured profiles between 20 mm and 36 mm (*z* distance) are shown in Figure 10.

**Figure 11:**
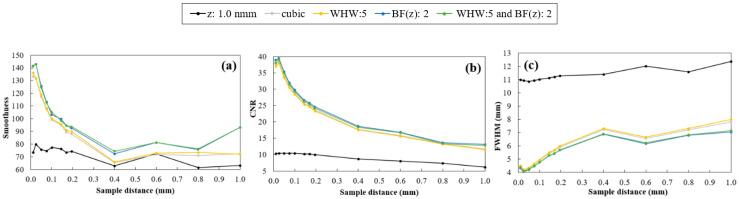
Smoothness (**a**), CNR (**b**) and FWHM (**c**) plotted as a function of sampling distance values for original data and data after interpolation with cubic, Hamming with WHW = 5, Hamming with BF (z) = 2 and Hamming with WHW = 5 and BF (z) = 2. Results obtained for rendered images at 90°.

For a quantitative analysis of the quality of rendered images, smoothness, CNR, and FWHM values were calculated for each sampling distance and the results obtained with the original data and after interpolation are shown in Figure 11.

**Figure 12:**
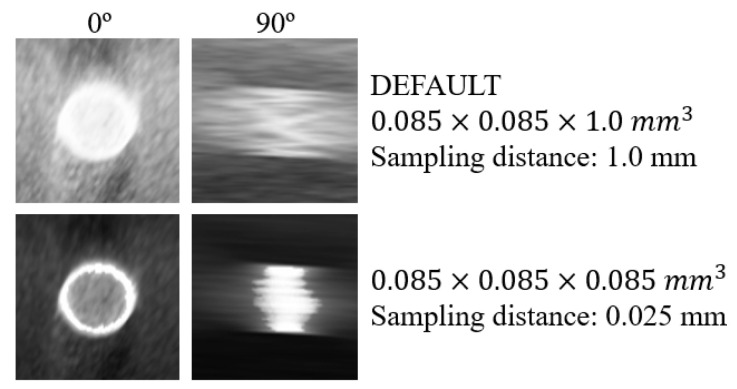
Volume rendering images at 0º and 90º for the 5-mm disk obtained for the original data with default visualization (top row) and interpolated data with Hamming window with BF (z) = 2 and sampling distance 0.025 mm (bottom row).

For qualitative inspection, sinc interpolation with Hamming window and BF (z) = 2 were selected and sampling distance of 0.025 mm were used. Images of the 5.0 mm disk of the phantom are presented in Figure 12. Images achieved with volume rendering at 0º and 90º are shown in the first and second column of Figure 12, respectively. The first row represents original data with default visualization (without interpolation and with sampling distance 1.0 mm), the second row presents “processed” data with Hamming BF (z) = 2 interpolation, and sampling distance 0.025 mm. The quantitative analysis corresponding to the images in Figure 12 is summarized in Table 3.

### 3.4. Clinical Data

**Figure 13:**
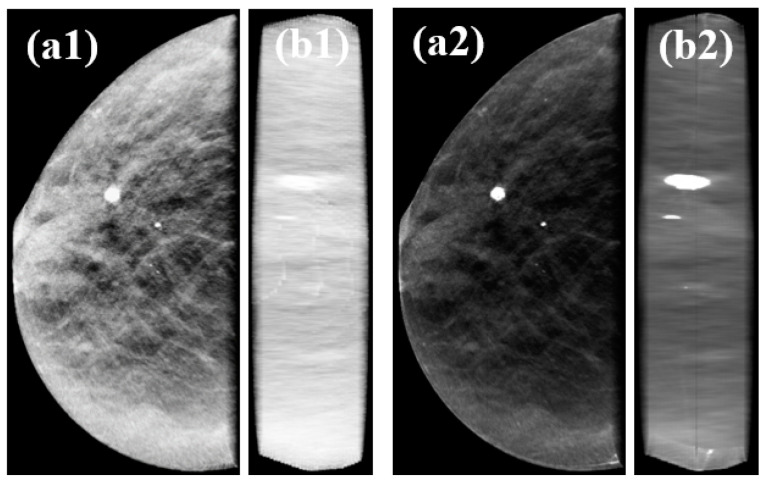
2D displays of composite volume rendering visualization obtained at 0º and 90º ((**a**,**b**), respectively) for original data with default sampling distance (1.0 mm) (**a1**,**b1**) and interpolated data with sampling distance 0.025 mm (**a2**,**b2**).

To evaluate the consistency of the results, sinc interpolator with Hamming window function with BF (z) = 2 was used in the rescaling of one clinical case (0.085 × 0.085 × 1.0 mm^3^ → 0.085 × 0.085 × 0.085 mm^3^). Volume rendering of clinical data was obtained with sampling distance value of 0.025 mm since it showed good results in terms of smoothness, CNR, and FWHM. 2D displays of composite volume rendering of clinical data obtained at 0° and 90° are shown in Figure 13. 

## 4. Discussion

This type of visualization is an alternative and complementary approach to the standard time consuming slice-by-slice visualization. Here, we optimized the volume rendering visualization for DBT data and our analysis was focused on two main parameters: interpolation methods used before rendering to obtain an isotropic grid (by modifying reconstructed voxel size); and sampling distance values.

For a constant sampling distance in the interpolation study, rendering time proved to be similar between the different interpolation methods (the minimum value recorded was 0.29 seconds for nearest and the maximum was 0.37 s for Nuttall) (Figure 3). On the other hand, when the number of voxels increases from approximately 8 million (0.085 × 0.085 × 1.0 mm^3^) to 95 million (0.085 × 0.085 × 0.085 mm^3^), a greater difference was observed. This means that changing the number of voxels had a greater impact on rendering time than the interpolation method itself.

Taking into account the profiles in Figure 4, with a smaller voxel size, a narrower profile was observed, confirming an improvement of image resolution. Among the three interpolators from *vtkImageInterpolator* class, nearest presented a profile with a blocky appearance resulting from the discontinuous interpolation between neighboring voxels. This translates into a lower smoothness value (greater variability) and a higher FWHM value (Table 3). Linear and cubic interpolators showed similar results for FWHM, with cubic showing a greater smoothness of the profile.

Window functions considered here (Lanczos, Kaiser, Cosine, Hann, Hamming, Blackman, and Nuttall) are already a selection of functions that produce high quality interpolations. Thus, by expecting a similar quality resultant from the different functions, our analysis was focused on two influential parameters: WHW and BF (z). By default, these options are set to 3 and 1.0, respectively, in VTK. Starting with WHW, there was a visible difference in the variability of the profiles in Figure 5, in particular until WHW = 5, with no major differences between interpolators. This observation was corroborated by the calculation of smoothness and FWHM values shown in Figure 6. In Figure 6a, there are six distinct groups, corresponding to the six WHW values tested and, for WHW values above 5, there was no noticeable increase in the smoothness value, increasing only the interpolation time. On the other hand, the variation of this parameter did not cause large fluctuations in the FWHM values for the different interpolators, as can be seen in Figure 6b. About the BF in z, it was found that this parameter has a great influence on the intensity fluctuations existent in the images. For example, from BF (z) = 1.0 (default) to BF (z) = 1.5 (Figure 7a,b, respectively), there was a significant modification in the profile of the disk at 90°. The results observed in the profiles were confirmed by the numerical analysis of smoothness (Figure 8a) which increases significantly for higher BF (z) values. It would be expected that the introduction of a blur parameter would increase the smoothness by compromising dispersion in the z-direction (increasing the FWHM value). However, as seen in Figure 8b, the introduction of this factor in the interpolation (up to a certain limit) helps delineating the structures during rendering. Until BF (z) = 2.5, there is a marked decrease in FWHM values for all window functions. Taking into account the temporal information of Figure 8a on the x-axis, we can see that these improvements were achieved with an increase in the interpolation time.

In Section 3.2, the selection considered for the sampling distance study has already been explained (WHW = 5, BF (z) = 2.0 and Hamming window function). While the choice of WHW = 5 was quite simple, for BF (z), we could have opted for 2.0 or 2.5. We selected the first instead of the second value because the interpolation time was shorter. As for the window function in sinc interpolator, it was not an obvious selection, since the different functions presented very similar results. In addition, for example, the Kaiser function has a parameter (α) responsible for the balance between blurring and ringing [31] and so, the effect of α on the results should be considered in a future work. Despite this and in view of the obtained results, Hamming function was the one with the best quality considering both WHW = 5 and BF (z) = 2.

By analyzing the time results obtained for various sampling distance values, if we compare Figure 3 and Figure 9, we found that sampling distance had much more impact on the rendering time than the number of voxels involved. Figure 9 shows an exponential curve with lower sampling distance values corresponding to much longer times. This was expected because sampling distance has a predominant role in the ray casting process.

In Figure 10, it is possible to notice once again the difference in the profiles obtained before and after rescaling (as it had already been observed before in Figure 5 and Figure 7 with sampling distance 1.0 mm). As for the interpolated data, in contrast to the profiles obtained with Hamming, the cubic interpolator showed some oscillation in intensity between 20 mm and 26 mm for all sampling distance values (Figure 10b). On the other hand, it is evident that the smaller the sampling distance, the greater the definition of the plateau, which corresponds to a well-defined disk in the rendered image. In Figure 11, the highlight goes to sampling distance of 0.025 mm, which shows a peak in smoothness and CNR and the lowest value of FWHM. In the same figure, taking into account the different sampling distances, the results for the four interpolations showed to be similar for CNR and FWHM. For the smoothness level of the profiles, Hamming with BF (z) = 2 and Hamming with BF (z) = 2 and WHW = 5 stood out, with the first one presenting the best results in general. All four interpolations lead to a significant improvement in the quality, when compared to the original data (black in Figure 11). This improvement is visible in the rendered images of Figure 12 where, particularly at 90º, the thickness of the disk can be observed with great definition (FWHM of 4.06 mm -Table 3), while with default values what we see is a blurred disk (FWHM of 12.38 mm -Table 3). This translates into a decrease of about 67% in FWHM values. Furthermore, there was an increase in CNR and smoothness of around 500% and 127%, respectively. At 0º, there is also an increase in CNR and a slight decrease in FWHM (about 4%). On the other hand, with interpolation and sampling distance of 0.025 mm, the time required for all the visualization process increases by approximately a factor of 10. Despite remaining at an acceptable value, time continues to be a crucial parameter in rendering and should be optimized.

Another viable option for the sampling distance could be 0.05 mm as it is 1.4 times faster than 0.025 mm, despite suffering some losses in image quality. Here is a summary of the numerical results obtained for the image quality with sampling distance of 0.05 mm (for comparison with the last column of Table 3): Total time: 2.19 s; CNR_0°_: 22.65; FWHM_0°_: 3.52 mm; CNR_90°_: 35.35; FWHM_90°_: 4.21 mm; and Smoothness_90°_: 125.7. Taking into account these results, for some applications the balance between image quality and time may compensate with a sampling distance of 0.05 mm.

A sampling distance of 0.025 mm and rescaling with Hamming BF (z) = 2 were applied to the visualization of a clinical data set. Rendered images of the clinical data (Figure 13) were in accordance with the results obtained with the phantom in terms of improving image quality, namely at 90°. There was a visual increase in contrast in the final image (Figure 13b) and the calcification present in this case had showed better defined contours. The preliminary clinical results are intended to consolidate the results obtained with the phantom. In the future, additional accurate studies with volume rendering of DBT clinical data should be considered to perform quantitative analysis of this type of visualization.

## 5. Conclusions

DBT visualization by volume rendering is a new field of research that may support breast cancer diagnosis. With this type of visualization, there is the advantage of observing the entire volume data set at once, from different angles. This is a complex process of visualizing volumetric data set, which includes several factors crucial for the final rendered image. The optimization of these factors is extremely important so that visualization through volume rendering can, along with 2D visualization, have true clinical value. In this way, the main objective of this work was to study and optimize two of these parameters: the interpolation used in the transformation of an anisotropic into an isotropic grid and appropriate sampling distance values, taking into account the entire time required. This is a very extensive study, so the analysis of other interpolation functions and their parameters should be considered in future work.

## Figures and Tables

**Table 1:** Summary of the functions and parameters, available in VTK, analyzed during the interpolation process.

In Study	
Image Interpolators	Linear
	Cubic
	Nearest-neighbor
Image sinc interpolators	Window Function (Lanczos, Kaiser, Cosine, Hann, Hamming, Blackman, Nuttall)

	Window Half-Width (WHW)
	Blur Factor in z-direction (BF(z))

**Table 2:** FWHM and smoothness values measured in interpolated data at 90º. The total time (interpolation plus rendering time) is also presented.

Interpolator	Linear	Cubic	Nearest
Total time (secs)	0.45	0.50	0.45
FWHM_90°_ (mm)	7.55	7.79	8.44
Smoothness_90º_	64.5	72.2	37.4

**Table 3:** Summary of the results obtained for phantom at 0° and 90°, with default visualization options (voxels with 0.085 × 0.085 × 1.0 mm^3^ and sampling distance 1.0 mm) and the options selected in our study (voxels with 0.085 × 0.085 × 0.085 mm^3^ after interpolation with Hamming window function and BF (z) = 2 and sampling distance 0.025 mm).

	Default	From Our Study
Voxel size (mm^3^)	0.085 × 0.085 × 1.0	0.085 × 0.085 × 0.085 (Hamming with BF (z) = 2)
Sampling distance (mm)	1.0	0.025
Total time (s)	0.23	3.05
CNR_0°_	7.19	22.12
FWHM_0°_ (mm)	3.67	3.52
CNR_90°_	6.23	39.39
FWHM_90°_ (mm)	12.38	4.06
Smoothness_90°_	63.0	142.8

## Abbreviations

2D	Two dimensional
3D	Three dimensional
BF	Blur factor
CNR	Contrast to noise ratio
DBT	Digital breast tomosynthesis
DM	Digital mammography
FWHM	Full with at half maximum
ROI	Region of interest
VTK	Visualization toolkit
WHW	Window half-width

## References

[1] Ferlay J., Colombet M., Soerjomataram I., Dyba T., Randi G., Bettio M., Gavin A., Visser O., Bray F. (2018). Cancer incidence and mortality patterns in Europe: Estimates for 40 countries and 25 major cancers in 2018. Eur. J. Cancer.

[2] Siegel R.L., Miller K.D., Jemal A. (2020). Cancer statistics, 2020. CA Cancer J. Clin..

[3] Berry D.A., Cronin K.A., Plevritis S.K., Fryback D.G., Clarke L., Zelen M., Mandelblatt J.S., Yakovlev A.Y., Habbema J.D., Feuer E.J. (2005). Effect of screening and adjuvant therapy on mortality from breast cancer. N. Engl. J. Med..

[4] Independent UK Panel on Breast Cancer Screening (2012). The benefits and harms of breast cancer screening: An independent review. Lancet.

[5] Poplack S.P., Tosteson T.D., Kogel C.A., Nagy H.M. (2007). Digital breast tomosynthesis: Initial experience in 98 women with abnormal digital screening mammography. AJR.

[6] Hubbard R.A., Kerlikowske K., Flowers C.I., Yankaskas B.C., Zhu W., Miglioretti D.L. (2011). Cumulative Probability of False-Positive Recall or Biopsy Recommendation After 10 Years of Screening MammographyA Cohort Study. Ann. Intern. Med..

[7] Gennaro G., Toledano A., di Maggio C., Baldan E., Bezzon E., La Grassa M., Pescarini L., Polico I., Proietti A., Toffoli A. (2010). Digital breast tomosynthesis versus digital mammography: A clinical performance study. Eur. Radiol..

[8] Brandt K.R., Craig D.A., Hoskins T.L., Henrichsen T.L., Bendel E.C., Brandt S.R., Mandrekar J. (2013). Can Digital Breast Tomosynthesis Replace Conventional Diagnostic Mammography Views for Screening Recalls Without Calcifications? A Comparison Study in a Simulated Clinical Setting. Am. J. Roentgenol..

[9] Bonafede M.M., Kalra V.B., Miller J.D., Fajardo L.L. (2015). Value analysis of digital breast tomosynthesis for breast cancer screening in a commercially-insured US population. Clin. Outcomes Res. CEOR.

[10] Gao Y., Babb J.S., Toth H.K., Moy L., Heller S.L. (2017). Digital Breast Tomosynthesis Practice Patterns Following 2011 FDA Approval: A Survey of Breast Imaging Radiologists. Acad Radiol..

[11] Destounis S., Santacroce A., Arieno A. (2017). DBT as a Screening Tool and a Diagnostic Tool. Curr. Breast Cancer Rep..

[12] Ramasundara S., Tucker L., Wallis M., Britton P., Moyle P., Taylor K., Sinnatamby R., Freeman A., Gaskarth M., Gilbert F. (2015). Diagnostic implications of digital breast tomosynthesis in symptomatic patients. BCR.

[13] Svahn T.M., Houssami N., Sechopoulos I., Mattsson S. (2015). Review of radiation dose estimates in digital breast tomosynthesis relative to those in two-view full-field digital mammography. Breast.

[14] Sechopoulos I. (2013). A review of breast tomosynthesis. Part I. The image acquisition process. Med. Phys..

[15] Hofvind S., Hovda T., Holen Å.S., Lee C.I., Albertsen J., Bjørndal H., Brandal S.H.B., Gullien R., Lømo J., Park D. (2018). Digital Breast Tomosynthesis and Synthetic 2D Mammography versus Digital Mammography: Evaluation in a Population-based Screening Program. Radiology.

[16] Simon K., Dodelzon K., Drotman M., Levy A., Arleo E.K., Askin G., Katzen J. (2019). Accuracy of Synthetic 2D Mammography Compared With Conventional 2D Digital Mammography Obtained With 3D Tomosynthesis. Am. J. Roentgenol..

[17] Van Schie G., Wallis M.G., Leifland K., Danielsson M., Karssemeijer N. (2013). Mass detection in reconstructed digital breast tomosynthesis volumes with a computer-aided detection system trained on 2D mammograms. Med. Phys..

[18] Iotti V., Giorgi Rossi P., Nitrosi A., Ravaioli S., Vacondio R., Campari C., Marchesi V., Ragazzi M., Bertolini M., Besutti G. (2019). Comparing two visualization protocols for tomosynthesis in screening: Specificity and sensitivity of slabs versus planes plus slabs. Eur. Radiol..

[19] Petropoulos A.E., Skiadopoulos S.G., Karahaliou A.N., Messaris G.A.T., Arikidis N.S., Costaridou L.I. (2020). Quantitative assessment of microcalcification cluster image quality in digital breast tomosynthesis, 2-dimensional and synthetic mammography. Med Biol. Eng. Comput..

[20] Food and Drug Administration (FDA) U.S Approval for software option 3DQuoromTM technology-Premarket Approval. https://www.accessdata.fda.gov/scripts/cdrh/cfdocs/cfpma/pma.cfm?id=P080003S008.

[21] 3DQuorum™ Imaging Technology—Improving Radiologist Performance through Artificial Intelligence and SmartSlices (White Paper). https://www.hologic.com/sites/default/files/downloads/WP-00152_Rev001_3DQuorum_Imaging_Technology_Whitepaper%20%20(1).pdf.

[22] Venson J.E., Albiero Berni J.C., Edmilson da Silva Maia C., Marques da Silva A.M., Cordeiro d’Ornellas M., Maciel A. (2017). A Case-Based Study with Radiologists Performing Diagnosis Tasks in Virtual Reality. Stud. Health Technol. Inform..

[23] Suetens P. (2009). Medical image analysis. Fundamentals of Medical Imaging.

[24] O’Connell A., Conover D.L., Zhang Y., Seifert P., Logan-Young W., Lin C.-F.L., Sahler L., Ning R. (2010). Cone-Beam CT for Breast Imaging: Radiation Dose, Breast Coverage, and Image Quality. Am. J. Roentgenol..

[25] Song H., Cui X., Sun F. (2013). Breast Tissue 3D Segmentation and Visualization on MRI. Int. J. Biomed. Imaging.

[26] Jung Y., Kim J., Feng D., Fulham M. (2017). Occlusion and Slice-Based Volume Rendering Augmentation for PET-CT. IEEE J. Biomed. Health Inform..

[27] Alyassin A.M. Automatic transfer function generation for volume rendering of high-resolution x-ray 3D digital mammography images. Proceedings of the Medical Imaging 2002.

[28] Alyassin A.M., Eberhard J.W., Claus B.E.H., Kaufhold J., González Trotter D.E., Kapur A., Pakenas W.P., Landberg C.E., Galbo C., Thomas J.A., Peitgen H.-O. (2003). 3D Visualization of X-ray Tomosynthesis Digital Mammography Data: Preference Study. Digital Mammography: IWDM 2002—6th International Workshop on Digital Mammography.

[29] Dharanija R., Rajalakshmi T. A Conjunct Analysis for Breast Cancer Detection by Volume Rendering of Low Dosage Three Dimensional Mammogram. Proceedings of the Progress In Electromagnetics Research Symposium Proceedings.

[30] Jerebko A., Engel K., Hofmann C., Mertelmeier T., Uchiyama N., Ongeval C.V., Steen A.V., Zackrisson S., Andersson I. 3D rendering methods for visualization of clusters of calcifications in digital breast tomosynthesis: A feasibility study. Proceedings of the ECR 2011.

[31] Preim B., Bartz D. (2007). Visualization in Medicine: Theory, Algorithms, and Applications.

[32] Schroeder W., Martin K., Lorensen B. (2006). The Visualization Toolkit: An Object-oriented Approach to 3D Graphics.

[33] Kitware (2010). The VTK User’s Guide.

[34] Siemens MAMMOMAT Inspiration-Tomosynthesis Option. https://www.accessdata.fda.gov/cdrh_docs/pdf14/P140011c.pdf.

[35] VTK Visualization Toolkit-VTK. http://www.vtk.org/.

[36] STEYX function. https://support.microsoft.com/en-us/office/steyx-function-6ce74b2c-449d-4a6e-b9ac-f9cef5ba48ab?ui=en-us&rs=en-us&ad=us.

[37] Thévenaz P., Blu T., Unser M., Bankman I.N. (2009). Chapter 28-Image Interpolation and Resampling. Handbook of Medical Image Processing and Analysis, 2nd Edition.

[38] VTK-Interpolators Visualization Toolkit-VTK-Interpolators. https://vtk.org/Wiki/VTK/Image_Interpolators.

[39] Nuttall A. (1981). Some windows with very good sidelobe behavior. IEEE Trans. Acoust. Speech Signal Process..

[40] Jähne B. (1995). 2.3.3. The Sampling Theorem. Digital Image Processing: Concepts, Algorithms, and Scientific Applications.

